# Activity and participation experiences of people with disabilities in Ethiopia

**DOI:** 10.4102/ajod.v11i0.1002

**Published:** 2022-09-16

**Authors:** Terry Krupa, Rosemary Lysaght, Yetnayet S. Yehuala, Heather M. Aldersey, Molalign B. Adugna, Dorothy Kessler, Beata Batorowicz, Jasmine Montagnese, Klodiana Kolomitro

**Affiliations:** 1 School of Rehabilitation Therapy, Queen’s University, Kingston, Canada; 2 Institute of Public Health, University of Gondar, Gondar, Ethiopia; 3 School of Kinesiology and Health Studies, Queen’s University, Kingston, Canada; 4 International Centre for the Advancement of Community Based Rehabilitation, Queen’s University, Kingston, Canada; 5 Department of Sociology, University of Gondar, Gondar, Ethiopia; 6 Faculty of Health Sciences, Queen’s University, Kingston, Canada

**Keywords:** Ethiopia, UNCRPD, ICF, capabilities approach, disability, inclusion, lived experience, qualitative research

## Abstract

**Background:**

Ethiopia, as a State Party to the United Nations Convention on the Rights of Persons with Disabilities (UNCRPD), has committed to upholding the rights of people with disabilities in Ethiopia. There is little evidence, however, reflecting the impact of this commitment on the lived experiences of people with disabilities in Ethiopia.

**Objectives:**

This study sought to uncover how the experiences of participation and activity shape the enactment of rights for Ethiopians with disabilities as enshrined in the UNCRPD.

**Method:**

Analysis of 25 qualitative interviews with people with disabilities and family members living in Ethiopia used a reflexive thematic analysis approach to arrive at central themes.

**Results:**

People with disabilities in Ethiopia experience marginalisation, distress and practical challenges in both routine daily activities and participation in broader social roles and opportunities. These experiences affect their ability to claim many of the rights afforded by the UNCRPD.

**Conclusion:**

Despite legislative efforts to bring about change in Ethiopia, people with disabilities continue to live on the social margins. A meaningful change will require substantial allocation of needed resources by the Ethiopian government to support national-level programmes and policy change. It is critical that people with disabilities and their families are engaged in receiving relevant support, and serve as change leaders.

**Contribution:**

This study illustrates how marginalisation, distress and practical challenges in daily activities and social participation arise and are sustained for people with disabilities in Ethiopia. The findings can help to inform the country’s efforts to enact the rights of Ethiopians with disabilities as enshrined in the United Nations Convention on the Rights of Persons with Disabilities.

## Introduction

According to the World Bank, 15% of the world population has experienced some form of disability, with most people with disabilities living in developing countries (World Bank Group 2018). The social status of people with disabilities in low- and middle-income countries has emerged as an international focus of interest, particularly since the release of the United Nations Convention on the Rights of Persons with Disabilities (UNCRPD) in 2007. The Convention seeks to improve the life experience and opportunities for people with disabilities by codifying a variety of basic social, economic, cultural, political and civil rights. As of 2021, 164 United Nations member states were signatories (United Nations [UN] Treaty Collection 2022). Ethiopia ratified the UNCRPD in 2010, meaning that it is legally binding in the country (UN Department of Economic and Social Affairs Disability 2022).

The impact of the Convention on the life experiences of people with disabilities is a central question underlying its practical importance. Venkatapuram (2014) considered the influence of the UNCRPD on both the legal changes observed in various countries in the aftermath of ratification, as well as the moral and ethical beliefs that foster such legislative change. What may be of greatest relevance, however, is how people with disabilities fare within local contexts. For example, a 2019 study conducted in three African countries (Kenya, Uganda and Zambia) by the United Kingdom (UK) Economic and Social Research Council and the UK Department for International Development aimed at examining the narratives of people with disabilities who experienced social and economic success, stories that they indicate are largely invisible from narratives about disability (Shakespeare et al. 2019). The study found very different legislative responses to the UNCRPD across these three nations, and a wide range of community assets in terms of educational opportunities, social supports and disability advocacy organisations. A key finding was that participants experienced many barriers and challenges, and because of limited assistance in the form of formal support structures, used their considerable personal resources (e.g. resilience, entrepreneurship and supportive social networks) towards successful participation outcomes (Shakespeare et al. 2019).

In Ethiopia, efforts have been made to document the prevalence of disabilities through surveys and national censuses. However, clear and reliable data trends are not apparent because of variations in the definition of disability used, which may have resulted in over- or under-reporting; moreover, prevalence is complicated by misconceptions about disability, lack of consistent data collection strategies across all regions of the country (especially rural areas) and unwillingness of families to reveal information about their children and family members (FMs) during data collection (Ministry of Labour and Social Affairs 2012). The World Health Organization (WHO) (2018) estimates that 17.60% of the Ethiopians live with some form of disability, suggesting that internal government estimates derived from ministry surveys and the national census (1.17% – 7.60%) have grossly underestimated its prevalence. Based on the demographic profile of the nation, a large percentage of these people live in rural settings. The current life expectancy for the general population is estimated to be 67.81 years (United Nations Data, 2022), with projections for ongoing increases in that number, suggesting that age-related disability will become a relevant issue in the years to come.

A number of factors challenge access to full social participation under the UNCPRD for people with disabilities in Ethiopia, including poverty, negative attitudes and stigma, infrastructure, policy, communication, and barriers arising from the physical environment (Getachew 2011; Tefera et al. 2015; Tekola et al. 2020). Several barriers have proven particularly problematic, such as harmful cultural and traditional practices, lack of proper childcare, civil war, chronic drought and famine, and absence of early preventive actions (Tefera et al.). Ongoing political instability and violence in the country have created conditions for increased disability in the population, and pose a challenge for efforts towards addressing the rights enshrined in the UNCRPD (Tesfaye & Mekuriya 2021). Poverty is perhaps the most significant barrier (Iyassu & McKinnon 2021), and coupled with rural living, leads to insufficient access to critical resources, including social services, health care and rehabilitation. People with disabilities are often expected to not only ensure an income for themselves but also provide for their families’ livelihoods (Franck & Joshi 2017). However, at the same time, they are believed to have little strength and to be unable to perform physical labour (Getachew 2011) and other demands of working, and many depend on family support and begging for their livelihoods (Franck & Joshi). The latter condition leads many people with disabilities to migrate from rural to urban areas, where begging is more profitable (Getachew 2011).

Disability is considered a highly taboo topic in Ethiopia, with attitudes often deriving from supernatural interpretations, frequently resulting in blaming and avoidance (Getachew 2011). Similar to the neighbouring country of Kenya, in Ethiopia disability is perceived to occur because of missteps by the mother, such as infidelity, or the way the mother treats others while she is pregnant (Bunning et al. 2017; Franck & Joshi 2017), thus, leading to the view that the child’s disability is a form of punishment or curse (Bunning). Despite the Ethiopian government’s efforts towards inclusive education, stigma continues to create barriers for students with disabilities and their access to education (Franck & Joshi). However, there has been limited progress towards implementing legal instruments of disability and inclusive education that might counter these views (Tefera et al. 2015).

The rights and opportunities provided to people with disabilities in Ethiopia vary in relation to several demographic factors. For example, individuals with mental disabilities can be restricted in exercising their legal rights to marriage, employment, property ownership and voting (Marishet 2017). Movements to challenge these restrictions are hampered by the lack of organised national advocacy efforts on behalf of, and including, people who live with mental disability (Abayneh et al. 2017). Disability experiences also intersect with gender. While generally more restricted in access to services and opportunities, the lived experiences of women with disabilities have only recently been a focus of the study (eds. Baron & Amerina 2007). A recent research study, for example, has demonstrated how the confidence, self-reliance and opportunities for women with disabilities who accessed higher education improved, but also had unintended consequences, such as dislocation from their places of birth and separation from their family (Tefera & Van Engen 2016).

Ethiopia has ratified a number of international instruments and treaties in addition to the UN Conventions, and the Constitution has made all international treaties ratified by the country ‘an integral part of the law of the land’ (Federal Democratic Republic of Ethiopia [FDRE] 1995). The treaties themselves oblige the State to take legal, institutional and practical measures. In 2012, as part of its commitment to adopt these international conventions, the Government of Ethiopia introduced the *National Plan of Action of Persons with Disabilities* (Ministry of Labor and Social Affairs 2012). This is a comprehensive plan aimed at mitigating the challenges and barriers faced by people with disabilities in every aspect of their lives. Ethiopia also launched a number of policies between 1994 and 2018, which address the needs for education, training, employment and building accessibility to improve the participation of Ethiopians with disabilities and ensure their basic and civil rights. The Constitution (Art. 41 [3 and 5]) upholds the rights of every Ethiopian national to equal access to publicly funded social services. It also articulates that support shall be provided to accommodate the needs of people with disabilities.

Despite the above efforts, the Constitution itself positions people with disabilities as dependent on others and refers to them as recipients of charity, suggesting that they are incapable of contributing to the development of the country. Art. 41(5) of the Constitution states that:

[*T*]he State shall, within available means, allocate resources to provide rehabilitation and assistance to the physically and mentally disabled, the aged, and to children who are left without parents or guardian. (FDRE 1995)

Furthermore, the 2016 report of the Committee on the Rights of Persons with Disabilities highlighted a number of concerns in areas, such as employment, civil and legal rights, and the accessibility of public services (United Nations, Committee on the Rights of Persons with Disabilities 2016). Problems with the operational definitions of ‘disability’ impact practical implementation of policy, including decisions related to focused, informed disability policy development, resource distribution and lack of coordination between state and non-state actors and human resource development, to name a few.

This study is grounded in two theoretical perspectives. The first is aligned with the International Classification of Functioning, Disability and Health (ICF) (WHO 2007). As a classification of health, the ICF identifies human activities and participation as important health-related states and recognises environmental and personal factors as contexts impacting health. In this way, the ICF looks beyond bodily functions and associated illnesses and injuries as the primary indicators of health. The activity and participation categories of the ICF identify many rights explicitly named within the UNCRPD, for example, education, work and employment, health, mobility, participation in public life, and the cultural, sports, leisure and recreation life of the community. Secondly, the study is grounded in the capabilities approach proposed by Sen (1999) and refined by Nussbaum (2003). This approach proposes that socio-economic development should advance human freedom. Capabilities are a form of freedom (Sen 1999), defined by ‘what people are actually able to do and to be’ (Nussbaum 2003:33). Stated simply, this approach focuses on the actions that support individuals to experience practical opportunities that they have reason to value. In this approach, the well-being and freedom of individuals are linked to the growth of human capital; the advancement of individual capabilities contributes to broader socio-economic well-being. Nussbaum proposed 10 central human capabilities to serve as a focus for the quality of life and social justice measurement for societies, these beginning with life, health, emotion and integrity, and more socially embedded elements, such as the right to affiliate with others, have control over one’s environment and to play. Sen (1999) observed that capabilities will vary by society, such that basic capabilities that should be guaranteed are determined within a local context. Both the ICF and the capabilities approach are concerned with human functioning; however, there are important distinctions. The ICF offers a descriptive classification system of functioning and disability, while the capabilities approach focuses on capabilities as essential to human well-being and equity. Disability scholars have suggested that there is a synergy between these two frameworks that may help to operationalise the capabilities approach (Bickenbach 2014; Mitra 2014).

In this study, the researchers sought to lay the groundwork for actions that could advance the socio-economic position of people with disabilities in Ethiopia. The study addressed the following research question: how do participation and activity experiences shape people with disabilities’ enactment of their rights as enshrined in the UNCRPD?

This study focuses on one aim of a needs assessment conducted in the Fall of 2019 and the Spring of 2020 to inform the development of a post-secondary occupational therapy education programme in northern Ethiopia. The goals of the needs assessment were to understand: (1) the activity and participation experiences of people with disabilities and those vulnerable to disabilities in Ethiopia, and (2) how occupational therapy as a profession might support presently unmet service needs related to disability in Ethiopia. While these are two distinct goals, they are highly related. The goals were purposely constructed to move the needs assessment beyond the biomedical perspective and approaches that have characterised Ethiopian health systems in order to enable understanding of the daily lived experiences of people with disabilities in relation to capabilities, functioning and practical opportunities as they are expressed within and influenced by the local context.

## Research design and methods

The full needs assessment involved surveys with 50 health service providers and stakeholders working in non-clinical roles (e.g. government and non-governmental organisation workers), and 44 interviews ith a range of stakeholders, including health service providers, non-clinical stakeholders, people with disabilities and their families. In order to study the activity and participation experiences of people with disabilities in Ethiopia, which is the focus of this study, we analysed the data collected during the 25 qualitative interviews with people with disabilities and their families. Ethical approval for the needs assessment, including both aims of the project, were received from the University of Gondar, Ethiopia (certificate #: O/V/P/RCS/05/354/2018) and Queen’s University, Canada (certificate #: REH-738-18). All participants provided informed consent, with consent forms and information about both goals of the project offered in their primary language.

### Recruitment of participants

People with disabilities and their families were recruited in two ways. Firstly, the directors of selected hospitals in Ethiopia accessed the hospital logbook of people with disabilities receiving services, and every other person listed in the logbook was invited to participate. Secondly, a purposeful selection of people with disabilities involved in post-secondary studies and supported through an inclusive education programme were approached to participate. Specifically, these participants were recruited with a view of ensuring a range of disabilities were included. Participants were included if they were aged 18 years or older, self-identified as a person with a disability (or a FM of a person with a disability) and could speak Amharic.

### Data collection

The interview questions were aligned with the ICF, in which activities are defined as the execution of tasks or actions by an individual, while participation refers to involvement in life situations (World Health Organization [WHO] 2001). The interviews were conducted by staff associated with the University of Gondar, who received training in qualitative interviewing and the goals of the needs assessment. Interview questions for people with disabilities in the community asked participants to reflect upon general activities, self-care and home living, community mobility and access, productivity, and leisure-social-recreation activities. Participants were also asked to reflect upon priority needs related to activity and participation, to identify barriers they face and to suggest solutions. The interview guide is presented in Table 1. Questions for FMs asked them to reflect upon these topics specific to their FM with a disability, as well as for their family more generally. Interviews lasted approximately 45 min – 60 min each and were conducted in Amharic. Interviews were transcribed in Amharic, and subsequently translated into English.

**TABLE 1:** Interview guide.

1. Personal questions about disability and living situation: Describe your health condition or disability. Describe your living situation.
2. Questions about experience of living with disability: Please describe a typical day for you, including your daily routines and habits. Thinking about your daily activities, are there some that are particularly meaningful and rewarding for you? In your experience living with your health condition or disability what have been the biggest challenges in your daily living? How do the activities you are doing now in your daily life compare with what you might like to be doing? Tell us about your experience with acceptance and inclusion in your community.
3. Questions about services and support received: Tell us about any services or formal supports you receive to assist you with your daily living. Tell us about any informal help or support you receive to assist you with your daily living. What kinds of services or supports would you find helpful in your daily life?
4. Final thoughts: Is there anything we have missed that you would like to tell us about living with disability?

### Data analysis

In order to examine the activity and participation of people with disabilities in Ethiopia (the focus of this study), analysis of the qualitative data followed Braun and Clarke’s (2006) six-phase reflexive approach to arrive at central themes. Consistent with Braun and Clarke’s method, the lens of analysis was grounded in the selected theoretical frameworks (i.e. capabilities approach and ICF) and immersion in the data (Braun & Clark 2021). Three of the current authors completed this inductive analysis, and subsequently every member of the research team involved in the development of the current study met on several occasions to reach agreement on interpretations. Themes were developed and integrated with direct quotes, and where differences in interpretation arose, investigators returned to the data.

Cross-cultural research is particularly vulnerable to difficulties in building the necessary rapport with participants and to misinterpreting the meanings of dialogue. In order to address these issues, translators were fluent in both languages, as well as with the key concepts and terms central to the focus of this study. Issues related to translation were brought back to the full investigative team and with team members having familiarity with the language and local cultural context enabling interpretation. Reflexivity was critical to analysis, with consideration provided to the extent to which interpretations were based on researcher assumptions. For example, where activities identified by participants were based in the local culture the research team expanded their understandings of the nature and context of these activities.

### Participants

All 25 transcripts conducted with people with disabilities and their families were analysed. These included 19 interviews with people with disabilities (8 females and 11 males) and six with FMs of people with disabilities (all female). Nine of the persons with disabilities experienced a physical disability-mobility impairment, six had a visual impairment, three lived with mental health issues and one had leprosy. All six FMs had FMs who experienced a physical disability or mobility impairment. A total of 10 out of the 19 persons with disabilities were university students, seven were unemployed (all formerly employed), one was retired and one was a homemaker. Seven participants were from Addis Ababa and the surrounding areas, nine from Gondar and the surrounding areas, two from Debre Markos town and the remaining from seven other geographical locations in Ethiopia (rural areas near Bahirdar, Deberebrehan, Telemet, Sendafa, West Harrerge, Dessie and Jimma). Interviews were completed in each of these regions.

The results are presented next with illustrative, direct quotes. The participants associated with each quote are identified by number and as either a person living with disability (PLWD) or a FM.

## Findings

The data suggest that people with disabilities in Ethiopia experience marginalisation, distress and practical challenges in both routine daily activities and broader social roles and opportunities. These challenges presented in various ways across the range of disability experiences, and thus differentially affected activity and participation patterns. For example, the study’s narratives included descriptions, such as this one, of people with disabilities who were largely disengaged from activities and participation, and idle and bored:

‘After I get sick [*disabled*], my leg, I could not move around because it was covered with jeso [*a bandage*] so I use crutches for movement. I only go for follow up. I stayed at home in the meantime. Due to this I could not participate in other activities so I feel frustrated. Well I still watch TV but I could not do any other voluntary activities. I perform all the activities through phone but not going out is what used to make me frustrated.’ (PLWD-11)

For those involved in social roles, such as being a student or parent, frustrations were common in response to both practical and social barriers to their full engagement in these roles. For example, PLWD and FMs alike spoke of having to give up employment when a disability was acquired. Post-secondary students in this study provided examples of being subject both to the negative attitudes of others who assumed that their academic accommodations were a form of unfair advantage, and of their grades being impacted by limited resources and learning processes designed to support their participation.

While there were many examples provided for receiving helpful instrumental and emotional support from others to enable activities and participation, the need for support could be experienced as a form of dependence and burden. For example, when FMs gave up work to provide care, this posed an economic strain on families and could damage relational bonds between FMs. One of the mothers stated:

‘Since I am not working my families consider me as a dependent person and sometimes they say offending words about me. I sold my house to take care of my child, I become below everybody. I was supposed to have better life. Now I am not working because I don’t have someone to look after my child.’ (FM-18)

Overall, the findings suggest that participants perceive a number of environmental barriers to full participation in activities fundamental to daily life. This situation was described as being sustained by several factors that ranged from a general lack of understanding of disability by the public to a lack of attention to the needs of people with disabilities in infrastructure planning to, at worst, discrediting the value of people with disabilities and their families. As a result, many people with disabilities and their families experienced feelings of shame, fear, unfairness and powerlessness. The situation also posed ongoing threats to subsistence that could lead to a choice to meet basic survival needs over activities that might provide meaningful and inclusive engagement. For example, participants spoke of needing to choose shelter over disability-related services and participating in begging as the only accessible way to earn an income.

As a result of this marginalisation and distress, people with disabilities expressed not being able to experience the health and well-being benefits that are associated with common activities and participation. Study participants observed the desire to contribute to personal well-being, their families and communities, particularly in the form of paid work. In addition to providing much needed income, the lack of access to work opportunities could result in a lack of structure to daily routines, and the loss of important social roles that provided identity and acceptance, and what one participant recalled as the ‘good life’.

Three themes emerged illustrating how marginalisation and distress arise and are sustained for people with disabilities: exclusion through attitudes about difference, disadvantage through infrastructure inequities and inhibited potential because of resource unavailability.

### Exclusion through attitudes about difference

Perhaps, the most pervasive social processes associated with limitations in the activity and participation patterns of people with disabilities in Ethiopia were those consistent with ‘othering’ – the casting of their roles in a society in damaging forms. This presented as disparaging people with disabilities through broadly held beliefs that disability is a form of supernatural curse. This belief, as illustrated by the following quotes, justifies marginalisation, shames the individual and the family, and damages social bonds:

‘But when I see the perception of others, even they do not think that disability is caused by different reasons. People think that disability is related to a curse or nature. For example, in our area, there are genes [*devils*] called “Angote” and “Eshetie.” That means “Angote” genes are not allowed to marry with the “Angote” themselves and the same goes for “Eshetie.” They associate disability with this and they think that the problem of disability comes as a result of injustice or sin of the family.’ (PLWD-42)

A university student stated:

‘They relate your disability with God’s blame and bad belief. Actually, in the beginning I also related it with bad belief before I came to this modern education. Even this was the reason that I was separated with my family because they associate it with this bad belief.’ (PLWD-14)

The marginalised status of people with disabilities is further sustained by the lack of awareness and understanding of disability among the general public. The participants described a range of public misperceptions about disability, including assumptions that the idleness experienced in the context of disability was evidence of moral failure. A lack of understanding was reported to be pervasive; a student with visual impairment stated:

‘Even educated people, including teachers, have a wrong view of disabled persons. As I’ve said, there is a problem that perceiving that all disabled people are the same.’ (PLWD-41)

Lack of knowledge was also evident through beliefs that disability was contagious, such that people did not want to touch a person with disability. As Participant 41 further observed:

‘In addition, there is a problem of understanding that if a person has a disability, they think that they will be exposed to other diseases. But I do not see blindness as exposing others to other disease.’ (PLWD 41)

With limited representation of people with disabilities fulfilling important social expectations and roles, such as earning an income, working and marrying, the prominent perception becomes one where individuals are viewed as incapable. One of the university students who had relocated from a rural to an urban area, for example, perceived that community members looked to participation in adult roles as evidence of capability:

‘There is lack of awareness in the society even if they see you can do it but they don’t want to admit it. It is actually better in the city but in the rural area they only say they can’t but not they can. Even when they see me learning they think that I am not capable. I think they expect this till I get married or get money and they don’t think I am learning here.’ (PLWD 15)

Participants also described societal attitudes that assumed that people with disabilities should not be expected to contribute and take on social responsibilities, and that this contributed to their segregation. One participant described how she became left out of her many previous community activities as a result of negative social responses to her disability:

‘Well previously I used to strongly participate in the community but now I am asking for help. I could not serve, or hold a position in idir [*i.e. an informal cultural community support network*] or female’s association like I used to be. I used to participate on different community development committees but I am now excluded in such areas. Even when there is election they say “please leave her, she is sick.” So, these things make me feel bad.’ (PLWD-13)

### Disadvantage through infrastructure inequities

Participants described how access to opportunities for activities and participation was made difficult in response to disabilities and observed a number of inequities in the social and physical infrastructure. Inequities in access to education negatively influenced the development of the knowledge and skills needed to pursue future jobs. One of the participants stated, ‘[*w*]hen we see education in rural areas it may be better when compared in the past, but still it is not enough, it is very low’ (PLWD 14). Another stated:

‘For example if there were two candidates with a degree for job vacancy; one blind and the other not. I think they will give support for those without disability. Actually now there is a thing to encourage people with disability on different medias, but I don’t think it is actually implemented so they give priority for healthy people with full potential. Therefore there need to be specific criteria for them.’ (PLWD 13)

Where work participation was impacted by disability, individuals and their families were highly vulnerable to economic insecurity, which, in turn, could lead to further limitations on work. For example, a farmer described how his inability to work as a result of a health condition led to the loss of farmland. There was a need for the affirmative development of work and income opportunities for people with disabilities in Ethiopia. It was suggested that while advancements in higher education for people with disabilities had been made, efforts in this regard had a limited focus (i.e. advantaging those with physical and vision-related disability), and there was a lack of attention to job opportunities upon graduation. One of the participants suggested that this lack of opportunities had led to begging as the default occupation for people with disabilities:

‘As I see from other places such as abroad there is an opportunity for them to live the life they want by themselves. So having this kind of organization in Ethiopia is good because there are a lot of people with disability who does not get support, some engaged in begging. So, if they get appropriate training on some jobs and get some skill, I am certain they can be independent.’ (PLWD-11)

Some participants expressed their views that common activities and participation limitations were not explicitly and routinely considered in the design, planning and implementation of community infrastructure. An urban-dwelling participant stated:

‘There is construction of many roads, buildings and a mall so not considering people with disability is lack of awareness. For example, when someone constructs a building there needs to be ramps for those with disability. I don’t think it will cost that much money when compared to the entire building.’ (PLWD-13)

This lack of attention to accessibility included problems related to locating important spaces (e.g. classrooms) in inaccessible locations, failure to include ramps and lifts, and the design of toilet facilities. In education and work environments, this included limited attention to availability of the tools or resources that would support participation, such as access to Braille books and other education and communication technologies:

‘[*W*]hen it comes to education there is a problem with materials. Still the problem exists. This has caused me problems. Not only for me but also for others. When you mention people with disability this problem is always mentioned. We cannot read any book we want. I still feel bad by this. The books are not prepared in Braille, or you cannot find them by audio or by softcopy. This one is another challenge. When we want to develop our mind there is no accessibility to meet our needs.’ (PLWD-14)

Participants observed the difficulties that the rural settlement patterns and rough terrain of Ethiopia posed to the activities and participation patterns of citizens, in general. These difficulties are magnified when it comes to integration of accessibility and inclusion in community and infrastructure planning. For example, a student who lives with a disability described how a desire to work in a rural area was limited by geography and road access:

‘I want to go to the rural area to teach but the main challenge is the landscape, there is water, mountains, low lands and the roads. So, the road problem must be solved – not only that – other infrastructures should be fulfilled.’ (PLWD-15)

Another participant perceived the extent to which she had to depend on the local community simply to leave her house because of the local climate and terrain:

‘I used to need support to move because my house’s landmark is very bad, it is downhill. Therefore, I need someone to hold me because I could not hold it with my hands. I was in bed for four months. I don’t go out. I was planning to sell the house because of this. The house is downhill, there is rain and mud so to pass that you need to have someone to support you. I just wait for the people to come. I don’t go out or come in as I want.’ (PLWD-13)

### Inhibited potential because of resource unavailability

The interview narratives provided descriptions of a range of factors that limit activity and participation, including unresolved issues with mobility, vision, strength, pain, stress and substance use management. Such restrictions could impact the ability to carry out everyday activities, such as basic self-care, lifting, household tasks and transportation use. That said, a primary concern was that people with disabilities and their families had inadequate access to the rehabilitation services, technologies, other health services and government supports that could make a positive difference in their lives. Consistent with the finding that disability is often afforded supernatural explanations, some participants described seeking cures through religious and traditional healing practices:

‘My son is now addicted to Khat [*psychoactive substance*]. He is having a mental health problem. He simply talks by himself but he does not do any other harm. We have tried a lot for his health with medical service and Tsiebel [*holy water*].’ (PLWD-11)

A participant from a rural area reported:

‘When I was a kid my rural family took me to a rural witch doctor and got me an operation to this [*tumor on the right side of his face near to his eye*]. Then when I grew up the wound was changed to another thing.’ (PLWD-12)

Where individuals did receive treatment from medical doctors and other health services, participants described limitations in the help they received. It was mentioned that they did not necessarily receive a clear explanation of their health condition and disability. Furthermore, there was the perception that reliable information from the health sector about disability was not reaching the broader public, and thus having little impact on the view of disability as a curse for wrongdoing. There was a problem observed with the quality of equipment available in the health and rehabilitation sector, specifically the breakdown of technology. The need for money to access treatment and disability-related equipment was reported as a barrier. One participant with hemiplegia noted the need for a wheelchair and assistive footwear, and asked, ‘I don’t have money because I don’t work. How can I buy a wheelchair for 60 000.00 Birr?’ (PLWD-9).

Beyond treatments directed to the health condition, there was an identified need for organised community services focused on promoting agency and a sense of well-being. A FM commented:

‘I can see how well she feels when someone comes and has a conversation with her. Therefore, it would be useful for us if we could get someone by our side who can visit us regularly and provide support and consult us about coping mechanisms that will make things easier.’ (FM-02)

Such health and rehabilitation services were considered important to the health and well-being of the family, as well as the individual with a disability:

‘It’s very important to know that people with disability are not the only one who need support and treatment; people who are giving care are also a victim for many things like stress, depression and discrimination. Therefore, the government should have different rehabilitation centers that give full accommodation for people with disability and the caregivers.’ (FM-02)

There was a significant need for agency and resourcefulness to advance their ability to engage in meaningful activities because of lack of community support:

‘Actually, it’s to do with my dream and to support myself. I participate in different associations and I get some benefits. You may take training. There may be some money you get during the training. I am also a musician, amateur musician. I used to work in night club and get some benefits. There were also some wealthy people and people who saw my goal in [*name*] town who supported me til high school and then til I took my 12th entrance exam.’ (PLWD-14)

The role of government and non-governmental organisations in providing disability-related services and access to opportunities for activities and participation in the community was highlighted as fundamental to meaningful change across the Ethiopian society. Respondents identified advocacy as a necessary precursor to change. It was observed that people with disabilities needed to be advocates through demonstrating what was possible, particularly in rural areas:

‘You need to show them that you can eat together. You need to show them by playing, joking through drama or doing other things … As I mentioned earlier most of Ethiopian population is found in rural area so we need to give awareness on that area, we people with disability.’ (PLWD-14)

It was noted that community members also need to help change attitudes:

‘Even if we people with disability are the one who need to take the lead, other educated community members need to change the awareness by using literature or other things, written or orally. The problem is big and it still exists.’ (PLWD-14)

## Discussion

The study findings highlight the barriers to activities and participation opportunities afforded to participants with disabilities in Ethiopia. These participants described the difficulties they experienced in accessing opportunities they perceive as meaningful, including work, school, involvement in activity groups, parenting and moving about their communities. Their experiences of these limitations include frustration, idleness and boredom, and a feeling of dependency.

While their descriptions observed the deprivations they experience in activity and participation in relation to impairments associated with disability, they also highlight the extent to which these are experienced as emerging from broader societal forces of marginalisation and exclusion. These forces include attitudes about disability, restrictions created by infrastructure inequities, and limited support services and resources. It is important to note that these marginalising forces are present even when participants are engaged in important activities, such as education and parenting. For example, students with disabilities in post-secondary studies, while experiencing impairments, such as blindness and mobility restrictions, study in contexts where access to learning resources, such as Braille books, is growing but still constrained; likewise, physical structures, such as stairs, continue to limit access to classrooms and deny access to other places where students and faculty might gather. These forces engender limitations in choice, lead to a focus on basic survival, and produce negative feelings, such as shame, fear, powerlessness and the sense of being infantilised. While there is the indication that such forces are perhaps stronger in rural communities, concerns were also raised that they were broadly relevant across the country. It is likely that such feelings of exclusion will be even more keenly felt in the post-war context based on the disproportionate impact of social unrest and regionalised disasters that is typically experienced by people with disabilities (Pineda & Corburn 2020). In addition, the population of people with disabilities has dramatically escalated because of the war, placing additional strain on existing resources (Berhe 2017).

From the perspective of the capabilities approach as developed by Sen and Nussbaum, the experiences of marginalisation and distress that were expressed provide evidence that people with disabilities in Ethiopia experience deprivations with respect to freedom to be able to perform the things they value, to develop their identities and potential, and to contribute to their communities. While it can be argued that many of these deprivations are experienced by Ethiopians more broadly, their impact on participation is perhaps more keenly felt in the context of disability. In fact, several participants with acquired disabilities reported how valued roles were lost as part of the transition to living with disability, and others described how they experienced deprivations even while engaging in valued social opportunities.

Nussbaum has suggested that jurisdictions can consider setting the minimal standards for capabilities, below which no citizen should fall, and thus identifying the rights to be ensured by governments and related institutions (Nussbaum 2003) and developed by concrete policies and actions (Comim 2014). In terms of physical accessibility, the Ethiopian Building Proclamation (FDRE 2009) seeks to regulate the construction industry in order to ensure that basic safety and quality standards are followed. Part of this code asserts the need for public buildings to be fully accessible to people with physical disabilities. This type of attention to disability access in public documents is important – but it is evident that widespread infrastructure change is slow to arrive because resources are limited.

Attitudinal change may be particularly difficult to address when beliefs are deeply enculturated. Stigma and discriminatory attitudes in Africa have been found to be prevalent in sustaining the marginalisation and exclusion of people with disability. In this study, examples were provided on how these attitudes could lead to both exclusion and segregation while individuals engaged in personally important activities. It was observed how these attitudes could be held by those holding important positions of authority (such as teachers, religious figures and government personnel) who might be expected to be in a position to both support an individual’s engagement and advocate for positive social change.

Disability awareness and advocacy will be instrumental for fostering empowerment, inclusion and development of people with disabilities in Ethiopia. Education will play a critical role in addressing the misperceptions that feed stigma, but positive contact between the public and people with disabilities fulfilling meaningful and socially valued roles has been demonstrated to be particularly impactful in changing attitudes and promoting acceptance (e.g. Chae, Park & Shin 2019; Novak, Feyes & Christensen 2011). Supports, legislation and opportunity structures will be needed to ensure that such contacts can happen.

Because of the economic constraints facing every system in Ethiopia (e.g. education, government, health care and employment), change will require creative solutions that are made in Ethiopia and draw on the strengths of the disability community itself. Community-based rehabilitation (CBR), an approach that is endorsed by the WHO, is already established in this country to address the needs of people with disabilities (see, for example, Asher et al. 2015; Fentanew et al. 2021). The core pillars of CBR include health, education, livelihood, social and empowerment (WHO 2010). Empowerment is perhaps the key element among these elements because of the apparent need for grassroots advocacy, and in order to address the sense of resignation that seemed present in many of our interviews. Rehabilitation professionals can assist in the change process by promoting access to needed services and by engaging people with disabilities in the advocacy process. As noted by Stevens et al. (2019:241), ‘rehabilitation professionals have the important job of allies and advocates for persons experiencing social restrictions in these domains as a result of stigma’. Given the strong biomedical approach currently informing the Ethiopian health system, it will be important to ensure that rehabilitation services are organised to champion participatory and empowerment-based approaches to service delivery.

### Limitations

This study was not without limitations. The study included a sample wherein a larger proportion of participants were university students and persons with visual impairments than in the general population of people with disabilities. While we made efforts to recruit across the country, most participants were from the northern region of Ethiopia. Readers are encouraged to take into account these participant demographics in considering the regional relevance of the findings of this study. Finally, interviews were conducted in Amharic. Although the study used rigorous processes for translation and back translation of data, some concepts may not have a perfect equivalency across the two languages of Amharic and English, and therefore, the study may have lost some of the original richness in description for an English language readership. We hope that the involvement of bilingual Ethiopians in the research team has helped to reduce the likelihood of misinterpretations. Despite these limitations, we believe that the study findings present valuable information on activities and participation as experienced by some people with disabilities in Ethiopia and their families.

## Conclusion

This study sought to uncover how participation and activity experiences shape the enactment of rights for Ethiopians with disabilities as enshrined in the UNCRPD. In this study, we have viewed the UNCRPD from the perspective of the activities and participation opportunities that people value, which have meaning in the local context. This may be useful in interpreting application of the Convention, in that activity and participation experiences can inform official data concerning disability, and help identify environmental factors, such as resources, social processes and infrastructure, that require attention in order to improve accessibility and acceptance.

The task of honouring the UNCRPD articles is a difficult one, particularly in a low-to-middle income country with many challenges to socio-economic development, while operating in a context of a civil war and pandemic. While many advancements have been made in achieving basic human rights for people with disabilities in Ethiopia since the country has become a signatory to the UNCRPD, much work remains to support capabilities, fully realise full human rights and enable full participation.

The study findings suggest a number of actions, which may assist in this transition, including the need for suitable physical and legislative infrastructure to ensure access to resources that support activity and participation, improved access to health and rehabilitation services, broad awareness raising to address social misperceptions of disability, and reinforcement and further development of disability organisations to support all of these actions, while also building empowering peer support networks for Ethiopians with disabilities and their families.
